# Shield Tunnel Crack Detection Based on Improved Unet

**DOI:** 10.3390/s26113360

**Published:** 2026-05-26

**Authors:** Gang Ming, Xiao-Wei Ye, Da Hang, Jian-She Qin, Jie Li

**Affiliations:** 1 Polytechnic Institute, Zhejiang University, Hangzhou 310015, China; 12212153@zju.edu.cn; 2 Department of Civil Engineering, Zhejiang University, Hangzhou 310058, China; cexwye@zju.edu.cn; 3 Hangzhou Metro Group Co., Ltd., Hangzhou 310017, China; qinjianshe@hzmetro.com (J.-S.Q.); lijie3@hzmetro.com (J.L.)

**Keywords:** shield tunnel, crack detection, DTA-Unet, dynamic convolution decomposition, triple attention

## Abstract

Unet, a deep learning architecture, has become one of the most widely used models for crack detection in the tunneling field. Although it performs well in overall crack image segmentation, it still has issues of limited feature expression capability and inaccurate segmentation. To address these problems, DTA-Unet was proposed based on dynamic convolution decomposition (DCD) and triple attention (TA). Firstly, the model used Unet as the baseline network and replaced traditional convolutions in the encoding-decoding process with DCD to enhance its feature extraction ability. Secondly, TA was combined with attention gate (AG) in the skip connections of the network, eliminating redundant information in spatial and channel dimensions to highlight the crack area. Finally, the proposed model was tested on crack datasets and compared with the conventional Unet model, image processing algorithms, and other deep neural network models in terms of detection performance on the datasets. The results show that it outperforms other advanced methods in crack detection performance. The proposed method is of significance to the maintenance of shield tunnel cracks.

## 1. Introduction

Concrete cracking is a common phenomenon in tunnels and can significantly affect the structural stability of tunnels [1,2]. Therefore, ensuring timely and accurate identification of cracks is essential for maintaining safe tunnel operation. However, conventional detection approaches usually depend on temporary tunnel closures for manual inspections [3], which not only increase economic costs and inspection time but also constrain detection accuracy. As a result, traditional methods are no longer sufficiently effective to satisfy the requirements of infrastructure maintenance.

Currently, there are two main methods for detecting tunnel cracks: image processing techniques and deep learning technologies. As digital image processing methods have continued to develop, numerous automated crack detection methods have been proposed, including edge detection algorithms [4,5], threshold segmentation [6,7,8], and directional variability [9,10]. Although these methods allow non-destructive monitoring, their effectiveness depends on image quality; moreover, feature extraction often still relies on manual participation, which in turn requires specialized knowledge. In contrast, deep learning has shown considerable potential for crack detection and has addressed multiple shortcomings of traditional methods [11,12,13]. Zhang et al. [14] compared three crack-classification approaches and found that convolutional neural networks (CNNs) provide clear advantages in both computational efficiency and detection accuracy, highlighting their suitability for this task. Likewise, Liu et al. [15] applied CNNs to the detection and four-category classification of cracks in infrastructure. Dorafshan et al. [16] conducted a comparative evaluation between CNN-based crack detection approaches and six widely used edge detection technologies. The results demonstrated that the CNN architecture outperformed the edge detectors. Additionally, Cha et al. [17] combined CNNs with a sliding-window strategy to realize patch-level crack detection, thereby enhancing the continuity of cracks in the detection results and improving detection performance. Subsequently, the Crack FCN model was proposed [18], which enabled pixel-level crack detection and substantially improved the accuracy of crack identification. In addition, several widely used semantic segmentation frameworks and their variants, including Unet [19,20], Faster R-CNN [21], and Mask R-CNN [22], have been extensively adopted in crack detection studies. Moreover, feature-based methods [23,24,25] have been developed to strengthen model detection performance through the incorporation of additional geometric descriptors. However, when applied to tunnel images collected under complex environmental conditions, these methods often lack sufficient robustness. As a consequence, tiny crack details are frequently lost, and crack segmentation is often incomplete. For this reason, there is an urgent need for semantic segmentation networks capable of adapting to complex tunnel environments to enable comprehensive and accurate crack identification.

To address these challenges, a crack identification framework, DTA-Unet (Dynamic Convolution Decomposition and Triplet Attention Unet), was proposed, using Unet as the foundational network. To enhance the model’s feature representation capability, all standard convolutions in the encoder–decoder process were replaced with DCD (Dynamic Convolution Decomposition). And a combination of Triplet Attention (TA) and Attention Gates (AG) was employed to refine the feature maps extracted by DCD. Subsequently, the model was trained using a crack dataset established from on-site inspections, and its crack identification performance was compared against image processing algorithms and other deep neural networks.

## 2. Crack Detection Model

### 2.1. DTA-Unet Model

For crack detection, extracting the morphological characteristics of cracks is essential for conducting subsequent safety assessments of the target structure. Therefore, performing semantic segmentation to identify crack morphology is more valuable for engineering practice than merely identifying image patches containing cracks. Pixel-level classification, enabled by fully convolutional networks, allows for crack segmentation and the identification of crack geometry. Unet is a prominent deep neural network architecture for semantic segmentation. It has been widely used as a backbone model for numerous researchers in various semantic segmentation tasks.

One of the significant challenges in training deep neural networks is the excessive number of parameters that require optimization. An abundance of trainable parameters necessitates a larger and more diverse dataset for effective model training; otherwise, the model risks underfitting or overfitting. Concurrently, a greater number of parameters imposes higher demands on hardware performance during training, leading to increased computational costs. For semantic segmentation tasks such as crack detection, which are essentially pixel-level binary classification problems (determining whether a pixel belongs to a crack or the background), enhancing the model’s expressive capacity is crucial. Before dynamic convolution was introduced, conventional convolutions used the same kernel parameters for all input images, which restricted the network’s capacity for feature representation. Typically, network depth or width is increased to boost expressive power, but this approach significantly escalates computational costs. Therefore, Yang [26] proposed a conditionally parameterized convolution in which an attention mechanism is assigned to multiple parallel convolutional kernels, breaking the characteristic of traditional convolutions that share parameters across all inputs. This method demonstrated excellent performance in detection, providing a new direction for enhancing model expressiveness. Building upon conditionally parameterized convolutions, Chen [27] introduced dynamic convolution, which accelerated model training and reduced the number of parameters. Li [28], analyzing the issues of large parameter counts in dynamic convolution and the difficulty in jointly optimizing dynamic attention with conventional kernels from a matrix decomposition perspective, proposed a Dynamic Convolution Decomposition (DCD) model incorporating a dynamic channel fusion mechanism. Compared to conditionally parameterized convolutions and dynamic convolutions, DCD features fewer parameters and improved performance. Consequently, this study replaces all standard convolutions in the encoder–decoder part of the Unet with DCD, aiming to achieve substantial performance gains with a relatively small increase in parameter count.

In deep neural networks, attention mechanisms assign varying weight parameters to input feature maps, enabling models to focus more on critical information while disregarding irrelevant details such as background. To facilitate information interaction across channel dimensions, the Squeeze-and-Excitation (SE) block was proposed [29]. However, the excitation phase primarily relies on dimensionality reduction and expansion operations, where reduction may hinder the effective learning of channel interdependencies. Wang [30] introduced an Efficient Channel Attention (ECA) mechanism, which eliminates dimensionality reduction and employs one-dimensional convolution to enable partial cross-channel interaction, thereby reducing the parameter count and improving performance. Liu [31] introduced a Normalization-based Attention Module (NAM), which leverages variance from batch normalization to represent the importance of channels and spatial locations, with greater variance indicating richer information and higher importance. Woo [32] proposed the Convolutional Block Attention Module (CBAM), integrating both spatial and channel attention mechanisms. While CBAM adaptively refines features and can be seamlessly integrated into CNN architectures, its spatial and channel attention components operate independently, lacking beneficial cross-dimensional interaction. Consequently, a nearly parameter-free Triplet Attention (TA) mechanism was introduced. It leverages residual connections and rotational transformations between tensors to achieve information interaction across three dimensions: channels, height, and width, while simultaneously avoiding the adverse effects associated with dimensionality reduction present in modules like CBAM. Compared to the aforementioned attention mechanisms, TA not only implements attention across spatial and channel dimensions but also facilitates cross-dimensional information exchange. Therefore, TA was selected for feature refinement. Furthermore, in crack identification, the background occupies a large area of the image, and the target cracks exhibit significant variations in shape and size. To address this, the present study combines TA with Attention Gates (AG). This combined approach not only enables the identification of cracks of various sizes but also mitigates the adverse effects of irrelevant regions and background information introduced by skip connections.

**Figure 1:**
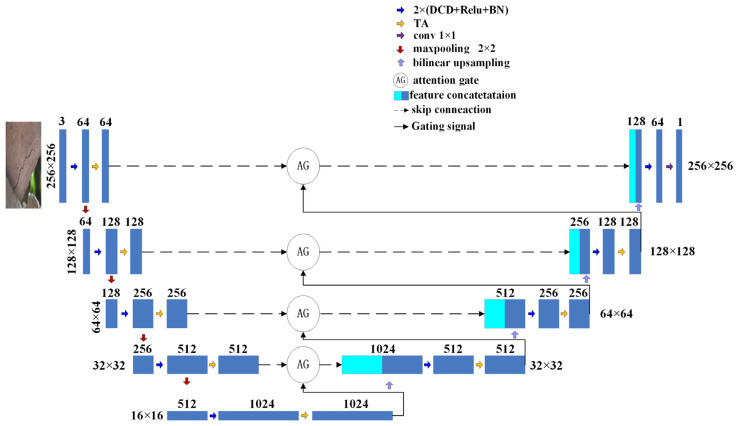
DTA-Unet Framework for Crack Detection.

The proposed crack identification network, DTA-Unet, is illustrated in Figure 1. It is built upon the Unet architecture, with DCD and TA incorporated into its fundamental network. First, a conventional encoder–decoder structure was employed in the network. The encoder consists of dual DCD blocks, TA, and max-pooling layers, responsible for feature extraction, feature refinement, reduction in image resolution, and increasing channel depth. The decoder comprises bilinear upsampling layers, dual DCD blocks, skip connections, TA, and 1 × 1 convolutions, tasked with restoring the encoded abstract feature maps to the original image size. Conventional CNNs are constrained by computational cost, limiting significant increases in network depth or width, which in turn restricts their expressive power. Replacing standard convolutions with DCD throughout the encoder–decoder process helps the network capture more detailed semantic information while effectively balancing computational cost and representational capacity. The fundamental principle of DCD is as follows:

(1) $$W(x)=\sum_{k=1}^{K}\pi_{k}(x)W_{k}$$

where *x* represents the input feature map, W(x) denotes the dynamic convolution kernel, $W_{k}$ signifies the standard convolution kernel, and k indicates the number of convolution kernels, with a value range of $\left[1,\;K\right]$. $\pi_{k}(x)$ refers to the attention weight coefficient, which ranges between 0 and 1. Additionally, $\sum_{k=1}^{K}\pi_{k}(x)=1$.

By leveraging residuals to represent each standard convolution, $W_{k}$ can be defined as:

(2) $$W_{k}=W_{0}+\Delta W_{k}$$

where $W_{0\;}=\;\sum_{k=1}^{K}W_{k}$ is the average convolution kernel.

(3) $$\Delta W_{k}=W_{k}-W_{0}=U_{k}S_{k}V_{k}^{T}$$

where $V_{k}$ signifies the right singular matrix, $S_{k}$ denotes the diagonal matrix, and $U_{k}$ is the left singular matrix corresponding to $W_{k}$.

Thus, the complete dynamic convolution is obtained as:

(4) $$W(x)=\sum_{k=1}^{K}\pi_{k}(x)W_{0}+\sum_{k=1}^{K}\pi_{k}(x)U_{k}S_{k}V_{k}^{T}=W_{0}+U\pi (x)SV$$

where *U* is the left singular matrix of $W_{\left(x\right)}$, with shape $U=\left[U_{1},\;U_{2},\;\cdots ,\;U_{K}\right]$. *S* is a unit diagonal matrix representing the convolutional weight coefficients obtained via the attention mechanism, with shape $S=\left[S_{1},\;S_{2},\;\cdots ,\;S_{K}\right]$. And V is the right singular matrix of $W_{\left(x\right)}$, with shape $V=\left[V_{1},\;V_{2},\;\cdots ,\;V_{K}\right]$.

If the constraint $\sum_{k=1}^{K}\pi_{k}(x)=1$ is relaxed, and the channel dimension is utilized to represent dynamic convolution, the channel count for a single standard convolution is $\begin{array}{l} C \end{array}$. Consequently, the total channel count for K standard convolutions becomes KC:

(5) $$W(x)=\Lambda (x)W_{0}+\sum_{i=1}^{KC}\pi_{i/C}(x)u_{i}s_{i,i}v_{i}^{T}$$

Finally, setting the dimensionality of the hidden space to L and letting L≪C, and substituting P, ϕ(x) and $R^{T}$ into the equation, the complete expression for DCD is expressed as:

(6) $$W(x)=\Lambda (x)W_{0}+P\phi (x)R^{T}$$

where *x* represents the input feature map. Both $W_{\left(x\right)}$ and $W_{0}$ are matrices of order $C\times k^{2}$, where C denotes the number of input channels, and $k^{2}$ indicates the convolution kernel size (k×k). $W_{\left(x\right)}$ denotes the Dynamic Convolution Decomposition. Λ(x) is a diagonal matrix of order C×C, generated by an attention mechanism. $W_{0}$ denotes the average convolution kernel. R is a matrix of order $k^{2}\times L$, designed to compress the number of kernel elements from $k^{2}$ to L. ϕ(x) is a matrix of order L×L, whose function is to dynamically fuse L elements, significantly reducing the dimensionality of the hidden space. P is a matrix of order C×L, used to increase the dimensionality back to the desired output channel count. To reduce the total number of parameters, the parameter L is manually set to $\left\lfloor k^{2}/2\right\rfloor$.

Furthermore, for further feature refinement, Triplet Attention (TA) is incorporated into both the encoder and decoder processes, enhancing the model’s focus on the segmentation target. Let the feature extracted by DCD be denoted as f:

(7) f=x⊙W(x)

where ⊙ denotes element-wise multiplication. The shape of f is C×H×W, where C represents the number of channels, H represents the height, and W represents the width.

**Figure 2:**
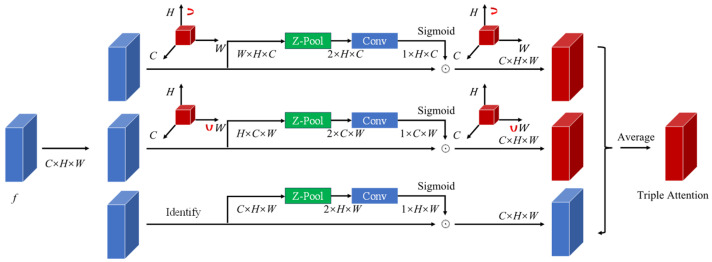
Calculation framework of TA.

The architecture of TA (Triplet Attention) is illustrated in Figure 2. A three-branch architecture was employed. The first two branches are similar, primarily utilizing rotation, Z-Pool, convolution, and Sigmoid functions to facilitate dimensional interaction between C&H and C&W, respectively. The third branch is designed to compute spatial attention weights. Finally, the outputs of the three branches are averaged. The resulting feature map not only incorporates the advantages of channel attention (CA) and spatial attention (SA) but also encompasses cross-dimensional interaction information, thereby achieving the objective of feature refinement.

The Z-Pool layer is responsible for reducing the C-dimensional tensor to 2 dimensions by concatenating the average-pooled and max-pooled features along that dimension. This allows the layer to retain a rich representation of the original tensor while reducing its depth, thereby further lowering computational intensity. The expression for Z-Pool is as follows:

(8) $$\text{Z-Pool}(f)=\left[Maxpool_{0d}(f),\;Avgpool_{0d}(f)\right]$$

where $Maxpool_{0d}(f)$ and $Avgpool_{0d}(f)$ denote the max pooling and average pooling operations applied along 0 dimension.

### 2.2. Loss Function

For binary classification tasks, the cross-entropy loss is commonly employed as the objective function for optimization, which is defined as:

(9) $$H(p,q)=-\sum_{i=1}^{k}p(i)\log q(i)$$

where *H*(*p*, *q*) represents the loss used for parameter updates, *k* denotes the total number of pixels, *p*(*i*) is the ground truth label for each pixel, and *q*(*i*) is the corresponding predicted value for each pixel.

The prediction for each pixel is generated using a sigmoid activation function, which is defined as:

(10) $$\sigma (z)=\frac{1}{1+e^{-z}}$$

where *z* denotes the value output by the model for each pixel. The output of the sigmoid function, *σ*(*z*), is bounded between 0 and 1, representing the probability that the pixel belongs to a given class.

### 2.3. Optimization Algorithm

This section employs the Adam optimization algorithm [33] to update the model parameters, which is expressed as:

(11) $$\theta_{t}\leftarrow \theta_{t-1}+\Delta \theta_{t}$$

where *t* and *t* − 1 represent the current and previous iteration steps, respectively. The symbol *θ* is the model parameter to be updated, and Δ*θ* refers to the parameter update increment, which is determined by the following equations:

(12) $$\Delta \theta_{t}=-\varepsilon \frac{\overset{\wedge }{s_{t}}}{\sqrt{\overset{\wedge }{r_{t}}}+\delta }$$

(13) $$\overset{\wedge }{s_{t}}\leftarrow \frac{s_{t}}{1-\rho_{1}}$$

(14) $$\overset{\wedge }{r_{t}}\leftarrow \frac{r_{t}}{1-\rho_{2}}$$

where *ε* is the learning rate, ${\overset{\wedge }{s}}_{t}$ and ${\overset{\wedge }{r}}_{t}$ are the bias-corrected first and second moment estimates, *δ* is a small constant added for numerical stability, and *ρ*_1_ and *ρ*_2_ are the decay rates for the first and second moment estimates, respectively. The first moment estimate $s_{t}$ and the second moment estimate $r_{t}$ are calculated according to the following formulas:

(15) $$s_{t}\leftarrow \rho_{1}s_{t-1}+(1-\rho_{1})g_{t}$$

(16) $$r_{t}\leftarrow \rho_{2}r_{t-1}+(1-\rho_{2})g_{t}^{2}$$

(17) $$g_{t}=\frac{1}{m}\nabla_{\theta }\sum_{i=1}^{m}L(f(x_{i};\theta ),y_{i})$$

In these equations, *g_t_* represents the mean gradient, *m* is the number of images in a training batch, *y_i_* is the ground truth label of a pixel, *L*(*f*(*x_i_*; *θ*), *y_i_*) is the loss function, and *f*(*x_i_*; *θ*) is the predicted output from the neural network.

### 2.4. Two-Stage Model Training Method

Concrete crack images captured in the field often contain complex background patterns, which can lead to identification errors even in trained deep neural networks. To this end, a two-stage model training method was proposed, which selects noise images without cracks.

After cropping and labeling crack images obtained during field inspections, this study constructed a dataset containing 6785 annotated crack images and 4285 noise images without cracks.

Manually selected noise images resembling cracks may reflect human bias and may not necessarily confuse a neural network. Training with such images does not substantially contribute to improving model robustness, so it is unnecessary to use all non-crack images for training. Moreover, training with larger datasets requires more powerful hardware resources. To enhance training efficiency, the following two-stage training method was proposed.

**Figure 3:**
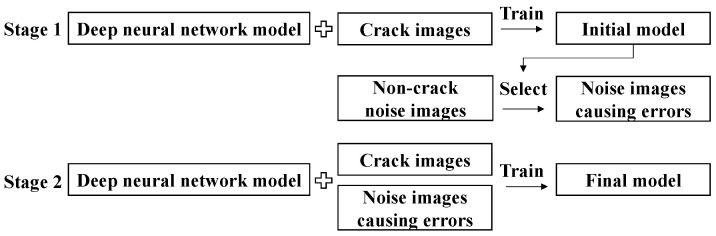
Two-stage model.

In the first stage, the deep neural network model is trained using only the 6785 pixel-wise annotated crack images, as shown in the Figure 3. Subsequently, the initially trained model is applied to process the 4285 non-crack noise images. Noise images that cause significant errors in the initial model are retained, while those that are easily distinguished are discarded.

In the second stage, the retained noise images are combined with the 6785 crack images to form an enhanced crack dataset. All subsequent network models are then trained using this consolidated dataset.

### 2.5. Model Evaluation Metrics

To evaluate the crack detection performance of the model, a total of five evaluation metrics were employed. These metrics are: Accuracy, Precision, Recall, Mean Intersection over Union (MeanIoU), and F-measure. They are defined by the following equations:

(18) $$Accuracy=\frac{TP+TN}{TP+FP+TN+FN}$$

(19) $$Precision=\frac{TP}{TP+FP}$$

(20) $$Recall=\frac{TP}{TP+FN}$$

(21) $$F_{\beta }=(1+\beta^{2})\frac{Precision\times Recall}{\beta^{2}\times Precision+Recall}$$

(22) $$MIoU=\frac{1}{j}\sum_{1}^{j}\frac{TP}{TP+FN+FP}$$

In these equations, TP refers to instances where the model correctly identifies a positive case, and the actual condition is positive. TN refers to instances where the model correctly identifies a negative case, and the actual condition is negative. FP refers to instances where the model incorrectly identifies a positive case when the actual condition is negative. FN refers to instances where the model incorrectly identifies a negative case when the actual condition is positive. *β* is a weighting coefficient that balances Precision and Recall, which is commonly set to 1. The variable *j* denotes the number of classes.

Accuracy reflects the ability of the deep neural network to make correct predictions across all classes. In semantic segmentation, correct classification includes background pixels. However, in crack detection tasks, the majority of pixels belong to the background rather than cracks. A model that predicts all pixels as background would achieve high accuracy, yet its actual performance in detecting cracks would be unsatisfactory. Precision indicates the proportion of instances predicted as positive that are actually positive. Recall represents the ratio of correctly identified positive instances to all actual positive instances. The *F_β_*-score is a weighted harmonic mean of precision and recall, where *β* is a predefined weighting coefficient. The MeanIoU measures the degree of overlap between the predicted positive regions and the ground truth positive regions.

## 3. Establishment of the Crack Dataset

### 3.1. Original Images

Deep learning is a representation learning method that enables a network model to learn relevant features and representations directly from raw data for subsequent classification tasks. However, the number of parameters requiring optimization in deep neural network models is exceedingly large. Consequently, for image-based crack identification tasks, a substantial quantity of crack images is essential for training the neural network. Furthermore, cracks in the field can manifest in various forms, characterized by differences in length, width, orientation, and other geometric attributes. More importantly, on-site crack images are often affected by various interfering factors, including variations in lighting conditions, water stains, rust, and surface markings. Therefore, an ideal crack dataset should comprise numerous crack images that encompass diverse geometric characteristics of the cracks themselves, as well as incorporate various types of noise patterns.

**Figure 4:**
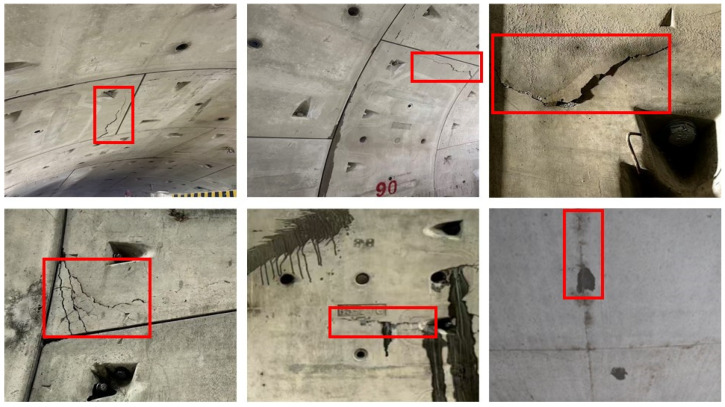
Typical Concrete Cracks.

To establish a crack dataset possessing the aforementioned characteristics, this study collected crack images from multiple projects through on-site photography, as shown in Figure 4. As the image acquisition was conducted by several teams, the collection task utilized multiple pieces of equipment, including smartphones, digital cameras, and DSLR cameras. The key parameters of the imaging equipment are detailed in Table 1. As shown in Table 1, the cameras used in this survey are representative of the types of equipment likely to be employed during routine inspections.

**Figure 5:**
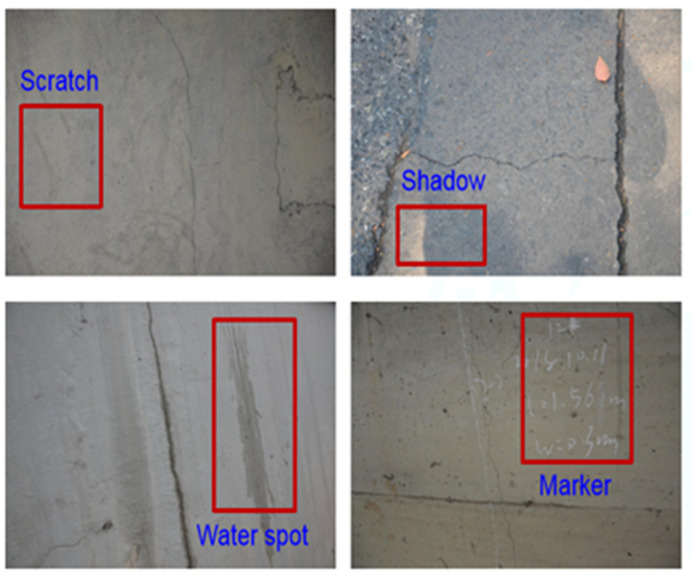
Original Crack Images.

Guided by the aforementioned objectives and methodology, a team of inspection engineers and researchers collected a total of 1600 raw crack images for use. To guarantee the quality of the initial image, the team performed a manual preliminary screening, discarding images captured under conditions such as poor focus or excessive exposure. Following on-site inspection and initial image screening, the team obtained 1300 structural crack images for establishing a concrete crack dataset. As illustrated in Figure 5, the raw crack images encompass not only various forms of structural cracks but also a range of noise patterns, including scratches, water stains, shadows, markings, and stains. To construct the structural crack dataset, the 1300 collected images of cracks were subjected to cropping and pixel-level annotation.

### 3.2. Image Cropping

**Figure 6:**
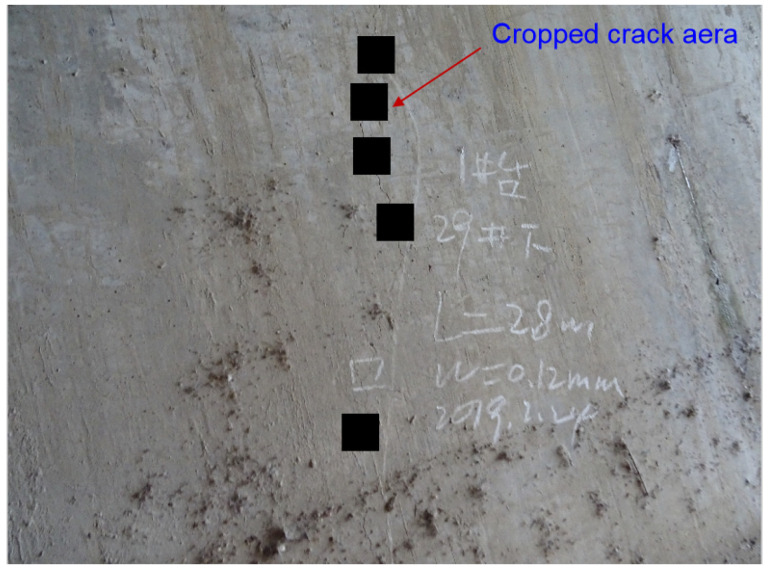
Process of Cropping Crack Sub-images.

Crack training images provide feature training samples for deep neural network models, enabling them to autonomously extract features to distinguish crack regions from the background. Therefore, the images used for training must accurately reflect the characteristics of cracks in the field, as well as the features of surrounding noise patterns. The morphology of typical cracks is generally slender and elongated, whereas nearby noise patterns, such as water stains, weld seams, or shadows, tend to be broader. Consequently, crack images must be of sufficient size to encompass both the complete crack region and its immediate surroundings. Otherwise, cropped images may only retain partial features of the crack area, thereby losing the capacity to represent both crack characteristics and the features of adjacent noise patterns. Considering the size of the original images, the geometric morphology of cracks, and the characteristics of noise patterns, the cropped image size was set to 256 × 256 pixels. Furthermore, this size can be readily down-sampled to smaller dimensions, such as 64 × 64 or 32 × 32, whereas the reverse process is considerably more difficult. A MATLAB program (version R2021b) was developed for this purpose, allowing an operator to capture images of the crack and its vicinity by clicking on the crack location on the screen. The process is shown in Figure 6. The image cropping task was performed by five personnel: two were responsible for selecting and cropping crack regions, while the other three served as independent image quality inspectors. Each cropped image was subjected to independent review by all three quality inspectors. A cropped sub-image was retained for subsequent processing only upon receiving unanimous approval from all three inspectors regarding its quality.

### 3.3. Image Labeling

**Figure 7:**
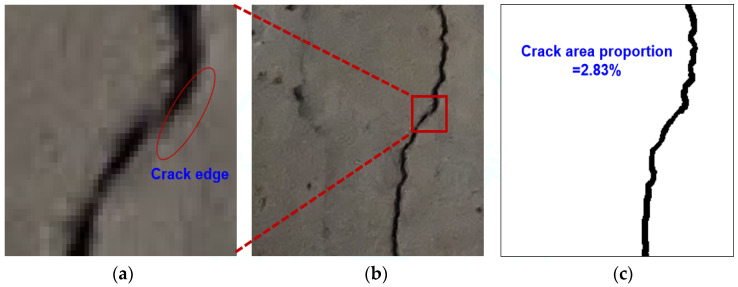
Crack Area Proportion: (**a**) Partial enlargement; (**b**) Crack image; (**c**) Image Labeling.

Within a crack image, the area occupied by the crack region typically constitutes only a very small portion of the total image area. As shown in Figure 7c, the crack region accounts for merely 2.83% of the entire image. Moreover, cracks are often very narrow, spanning only a few pixels in width. For example, the crack shown in Figure 7a has a width of approximately 3 to 8 pixels. Because the area occupied by cracks is extremely small, the performance of deep neural network-based crack identification is particularly sensitive to the accuracy of annotations for the crack regions within the training images. Therefore, for slender patterns like structural cracks, meticulous annotation, especially the correct delineation of edge regions, significantly impacts the training outcome of the deep neural network for crack identification. In contrast, during semantic segmentation tasks such as pedestrian or vehicle detection, the target objects often occupy a substantial portion of the entire frame. In such cases, inaccuracies in edge annotation tend to have a diminished effect on the final detection performance.

**Figure 8:**
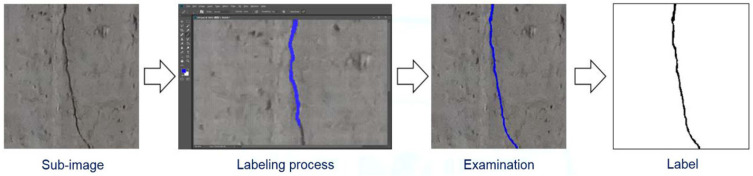
Illustration of the Annotation Process.

The image annotation process was implemented using Photoshop software (version 22.1.0). The annotation process is shown in Figure 8. Prior to annotation, the target image with a resolution of 256 × 256 pixels was enlarged on the computer monitor to provide a clear view of crack details. During crack annotation, the stylus was used on the tablet to trace the crack region as precisely and completely as possible, with particular attention paid to distinguishing between crack edges and the background. Similarly to the quality control measures applied during image cropping, the annotation task was performed by two operators and subsequently inspected by three independent image quality reviewers. If any reviewer rejected the annotation, the image was re-annotated. Regarding time consumption, the initial annotation of a single 256 × 256 image typically required approximately 2 min. The subsequent quality inspection took about 30 s. If the initial annotation quality was deemed insufficient and re-annotation was necessary, this re-annotation process required approximately 1 min. Consequently, obtaining a satisfactorily annotated crack image took roughly 2.5 min under optimal conditions. If the first attempt failed, the total time increased to at least 4 min. For annotating non-crack regions, as this dataset focuses on a binary classification problem, assigning the entire non-crack area as the background was a straightforward process.

**Figure 9:**
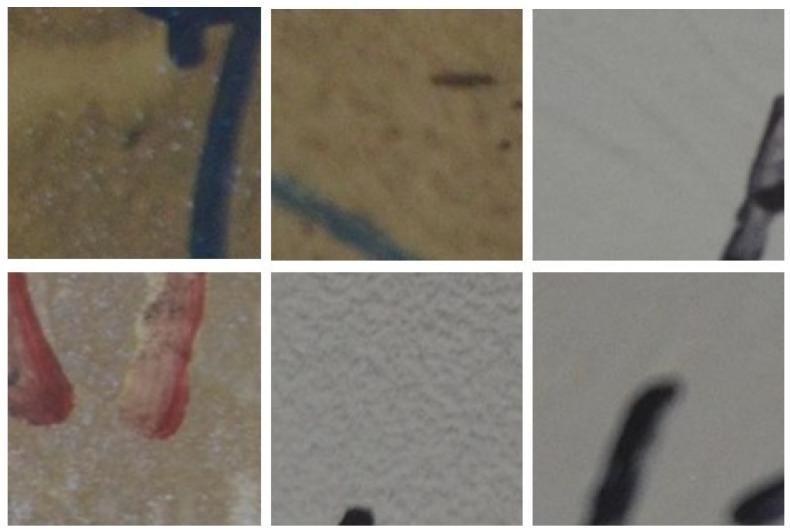
Examples of Noise Images.

Based on the aforementioned procedures, this section ultimately obtained 6785 crack images with a resolution of 256 × 256 pixels and pixel-level annotation, derived from the originally collected concrete crack images. Additionally, 4285 images containing similar crack-like patterns were retained. Representative examples of the retained noise images are presented in Figure 9.

**Figure 10:**
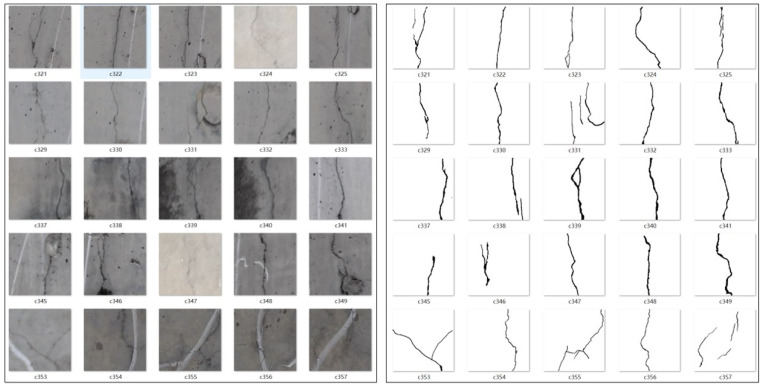
Concrete Structural Crack Dataset.

Upon completion of the aforementioned steps—collection of original images, cropping of images, annotation of images, and filtering of noise images—a concrete crack dataset was established. This dataset comprises 11,070 images with a resolution of 256 × 256 pixels and pixel-level annotation. Among these, 6785 are crack images, and 4285 are noise images. Representative examples of crack images alongside their corresponding annotations are presented in Figure 10.

## 4. Model Training

To identify the optimal model that balances performance and computational efficiency, four training configurations were selected, as detailed in Table 2. The basic model refers to the Unet deep neural network [20]. Cases 1–2 correspond to the first-stage training, where the training dataset consisted solely of 6785 crack images with pixel-wise annotations, excluding any non-crack noise images. Cases 3–4 represent the second-stage training. The training set for these cases included the 6785 annotated crack images along with the challenging noise images filtered by the initially trained model from the first stage. These were the noise images that induced a higher rate of identification errors. By learning the features of these highly interfering noise images, the deep neural network can improve its robustness against various disturbances and enhance the accuracy of crack detection. In this study, the Adam optimizer was configured with the following hyperparameters: a learning rate of 0.001, a learning rate decay factor of 0.1, a batch size of 32, a momentum value of 0.5, and training was conducted for 200 epochs.

The training of the deep neural network models was conducted on a high-performance server. The hardware and software specifications of the server are listed in Table 3.

For Stage 1 training, the 6785 crack images were split into a training set and a validation set. A total of 6100 crack images were allocated for training, while the remaining 685 crack images were used for validation, resulting in a training-to-validation ratio of approximately 9:1. To evaluate the model’s robustness in crack detection, an additional 210 manually selected noise images from the pool of 4285 noise images were added to the validation set. Consequently, the final validation set used for calculating the five evaluation metrics consisted of 895 images, each with a resolution of 256 × 256 pixels. During the 200 training epochs, the model with the best Mean Intersection over Union score on the validation set was saved for subsequent crack detection evaluation. The performance of each model after Stage 1 training is presented in Table 4.

**Figure 11:**
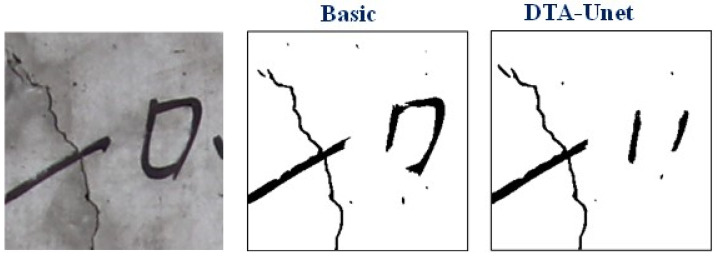
Crack Detection Test in Stage 1.

As shown in Table 4, models trained exclusively on crack images achieved Accuracy rates above 0.97; however, the Precision, Recall, and F-measure scores remained relatively low. Although DTA-Unet demonstrated better MeanIoU performance, its score was only 0.4411, which is insufficient for crack detection requirements in engineering applications. This is because the validation set contained numerous noise images located away from cracks, which were not encountered during training, leading to misidentification by the models. To visually assess the crack detection performance of the model trained in Stage 1, image-based testing was conducted, and the results are presented in Figure 11.

As shown in the crack detection tests in Figure 11, dark-colored pen marks and some small spots were incorrectly identified as cracks. The initially trained deep neural network models still struggled to accurately determine whether dark patterns represented structural cracks. The basic model was more susceptible to the influence of noise patterns, resulting in a higher number of misjudgments caused by spots on the concrete surface. The crack detection performance reflected in Figure 11 demonstrates that models trained solely on 6785 crack images have insufficient identification capability, and their results are prone to errors due to noise interference. Since concrete surfaces often contain numerous noise patterns, such as marks and spots caused by various factors, these can lead to incorrect identifications. Therefore, incorporating noise images into training can significantly reduce such interference, which is of critical importance for engineering applications.

**Figure 12:**
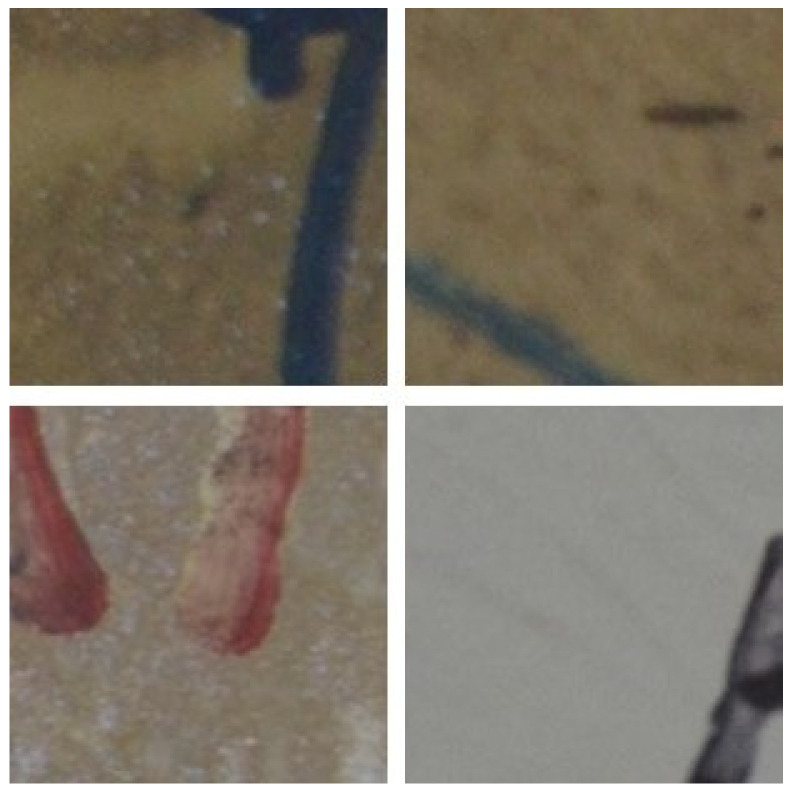
Retained Noise Images.

Based on the experimental results described above, the initially trained model was used to process the remaining 4285 non-crack images with a resolution of 256 × 256 pixels. According to the identification results, noise patterns that caused strong interference were retained. After this processing, 3000 images with higher interference levels were preserved, while the remaining non-crack images with lower interference were discarded. Representative examples of the retained non-crack noise images are shown in Figure 12.

After retaining 3000 highly interfering non-crack noise images, a crack dataset containing a total of 9785 images was established. For Stage 2 training, both models were trained using the complete set of 9785 images. To ensure consistency in evaluation, the validation set remained the same 895 images used in Stage 1. The evaluation metrics obtained from training under this setting are shown in Table 5.

**Figure 13:**
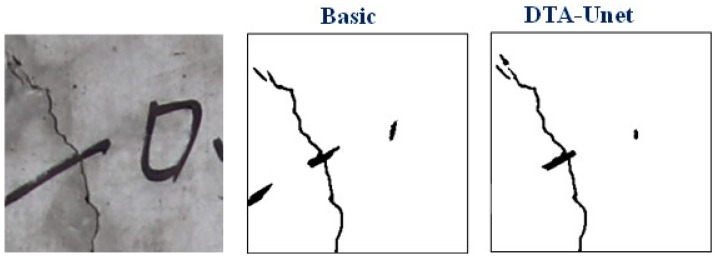
Crack Detection Test in Stage 2.

As shown in Table 5, after incorporating non-crack noise images into model training, all five evaluation metrics showed significant improvements. MeanIoU is a key evaluation metric for crack detection. As shown in Table 5, both models achieved MeanIoU values above 0.5, indicating satisfactory detection performance. Compared with Stage 1, the MeanIoU, Precision, Recall, Accuracy, and F-measure increased numerically by 0.1674, 0.2359, 0.0582, 0.0091, and 0.1437, respectively, representing relative improvements of 37.60%, 40.08%, 9.01%, 0.93%, and 23.32%. Since crack regions constitute relatively small targets within crack images, and the majority of pixels belong to the non-crack background, the improvement in Accuracy was limited, while the other metrics improved significantly. Based on the evaluation metrics, the DTA-Unet model outperformed the other models in all five metrics: MeanIoU, Precision, Recall, Accuracy, and F-measure. Crack detection was performed using the models from this stage on the same set of images used in Stage 1, and the results are shown in Figure 13.

Overall, compared to the models obtained from Stage 1 training, both models can correctly segment crack regions in the presence of various noise patterns such as markings. This demonstrates that the crack detection capability of the models has significantly improved after incorporating non-crack noise images. However, both models still misidentify a small number of handwriting traces as cracks. Based on the metrics and the detection results, the DTA-Unet model performs better between the two, exhibiting fewer misidentifications during the detection process.

## 5. Comparison of Different Crack Detection Methods

### 5.1. Comparison with Image Processing Algorithms

In previous studies, image processing algorithms have also been employed for crack detection. Three commonly used image processing algorithms for crack detection were employed, and their performance was compared with the DTA-Unet model. The three algorithms are the Canny algorithm, the LOG algorithm, and the Sobel algorithm. The same five evaluation metrics are applied here, and the performance comparison is presented in Table 6.

As shown in Table 6, compared to DTA-Unet, the three image processing algorithms perform significantly worse in all metrics except for Accuracy, which remains relatively close. This discrepancy can be attributed to the complex morphology of cracks and the significant variations in crack images under different lighting conditions, which reduce the robustness of image processing algorithms. Furthermore, crack images often contain various noise interferences such as water stains, scratches, and markings, making it difficult for the segmentation methods commonly used in image processing algorithms to effectively distinguish crack regions.

### 5.2. Comparison with Other Deep Neural Network Models

To evaluate crack detection performance, the DTA-Unet model was compared with other deep neural network models. A fully convolutional neural network (FCN) model based on the VGG architecture [34] and the Deeplab V3 model [35] was employed for pixel-level crack detection comparison. The performance metrics of DTA-Unet and these two models are presented in Table 7. Overall, among the three models, DTA-Unet demonstrates the best crack detection performance.

**Figure 14:**
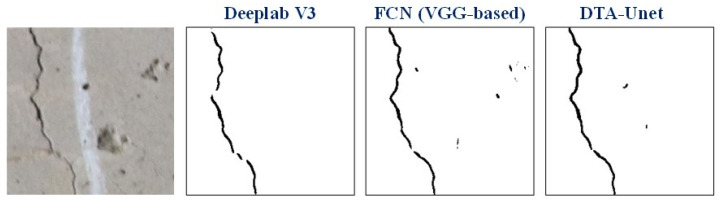
Crack Detection of DTA-Unet and Other Deep Neural Network Models.

The image-based comparison among DTA-Unet and other models is illustrated in Figure 14. Overall, all three deep neural network models successfully identified structural cracks on the concrete surface. White markings were effectively excluded. However, the crack regions identified by Deeplab V3 appear less continuous compared to those identified by the other two models, and the results reflect a more conservative tendency, consistent with the metrics shown in the table. Additionally, stains in the figure caused some interference in crack detection, with both the VGG-based fully convolutional neural network and DTA-Unet making incorrect judgments to varying degrees. Considering both Table 7 and Figure 14, DTA-Unet demonstrates superior crack detection performance.

**Figure 15:**
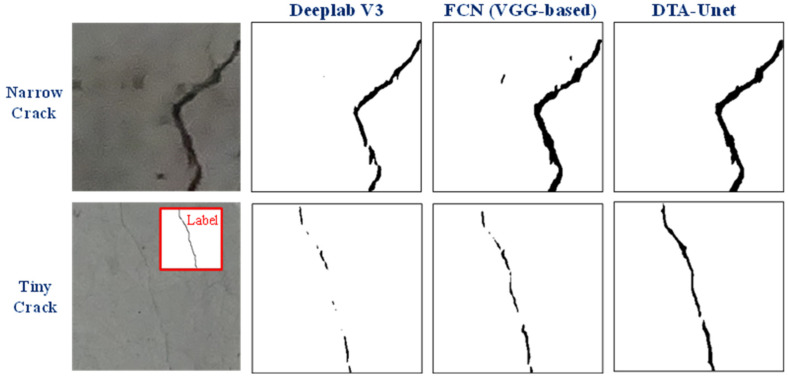
Crack Detection Test on Cracks of Varying Widths.

In order to investigate the crack detection capabilities of the three models mentioned above, image tests on cracks of varying widths were conducted, as shown in Figure 15. The results for detecting the wider crack illustrate the conservative nature of Deeplab V3. Although it preserved the overall shape of the crack, compared to the other two models, it failed to capture portions of the crack area, resulting in lower completeness of the crack’s geometric continuity. The detection results for the tiny cracks further highlight this conservatism in Deeplab V3. While adopting a conservative prediction strategy can help resist noise and reduce false judgments, it may also cause the model to overlook cracks with smaller widths. The VGG-based FCN detected some parts of the crack regions, while DTA-Unet identified more complete crack areas. Thus, in terms of detecting cracks of varying widths, DTA-Unet again demonstrates stronger performance.

## 6. Conclusions

In this study, a deep neural network model named DTA-Unet was proposed, developed through model modification and a two-stage training strategy. Based on the crack dataset established in this study, the crack detection performance of the model was compared with three image processing algorithms and two other deep neural network models. The conclusions are as follows:

- The established dataset contains a rich collection of crack samples and noise samples. Therefore, it can be used to train deep neural network models with high robustness for crack detection, and can also serves as a benchmark for evaluating the performance differences among network architectures in crack detection tasks.
- By replacing the conventional convolution kernels in the original Unet with dynamic convolution kernels, crack features can be preserved to the greatest extent possible during channel reduction. Moreover, after dynamic convolution is applied, spatial and channel information can be immediately exchanged through the TA structure. This not only enhances the feature representation capability of the original architecture but also reduces interference from regions unrelated to cracks, thereby yielding crack recognition performance markedly superior to that of the original architecture. Compared with Unet, the MeanIoU increased by 0.0312, corresponding to a relative improvement of 5%.
- By employing the two-stage training method, the DTA-Unet model achieved improvements of 37.60%, 40.08%, 9.01%, 0.93%, and 23.32% in MeanIoU, Precision, Recall, Accuracy, and F-measure, respectively. Collecting more crack images from engineering environments with various noise types is crucial for enhancing the performance of deep neural network models in crack detection and for engineering applications.
- In terms of crack detection performance, image processing algorithms are more susceptible to noise patterns and are more prone to erroneous judgments. In contrast, deep neural network-based methods offer better robustness and higher accuracy. The comparison between DTA-Unet and the other two deep neural network models demonstrates that DTA-Unet provides more accurate crack identification.
- Although the model has demonstrated the ability to identify cracks in the images collected in this study, the current dataset, which includes images with different crack shapes, crack sizes, and noise patterns, remains insufficient to guarantee its applicability to all types of tunnel projects. For crack types or noise patterns not represented in the training dataset, effective identification may not be achievable. Since the primary purpose of this model is to achieve more efficient identification of tunnel cracks, practical engineering applications require rapid and accurate crack detection. Before practical application, the network needs to be made more lightweight in order to improve its overall recognition efficiency.

## Figures and Tables

**Table 1:** Photography Equipment.

Brand	Model	Sensor Type	Sensor Size	Resolution
Canon	EOS 77D	CMOS	22.3 mm × 14.9 mm	6000 × 4000
Canon	EOS 800D	CMOS	22.3 mm × 14.9 mm	6000 × 4000
Nikon	D5100	CMOS	23.6 mm × 15.6 mm	4928 × 3264
Sony	DSC-HX60	CMOS	1/2.3”	5188 × 3888
Vivo	X23	CMOS	1/2.55”	4035 × 3204

**Table 2:** Four training configurations.

Case	Model	Dataset
1	Basic model (Unet)	6785 crack images
2	DTA-Unet	6785 crack images
3	Basic model (Unet)	6785 crack images + DNN-filtered strong noise images
4	DTA-Unet	6785 crack images + DNN-filtered strong noise images

**Table 3:** Hardware and Software Specifications of the Training Setup.

Platform	Component	Specification	Model Number
Server	hardware	CPU	2 × Intel(R) Xeon(R) Silver4215R CPU@3.20 GHz
		GPU	NVIDIA RTX 3090/GDDR5X 24 GB
		RAM	64 GB
	software	Windows 10 Professional	
		Pytorch 1.7.1	
		Python 3.8.5	
		Opencv 4.4.0	

**Table 4:** Performance Comparison of Models After Stage 1 Training.

Model	MeanIoU	Precision	Recall	Accuracy	F-Measure
Basic model	0.4047	0.5442	0.6123	0.9775	0.5762
DTA-Unet	0.4452	0.5886	0.6463	0.9798	0.6161

**Table 5:** Performance Comparison of Models After Stage 2 Training.

Model	MeanIoU	Precision	Recall	Accuracy	F-Measure
Basic model	0.5814	0.7852	0.6913	0.9875	0.7353
DTA-Unet	0.6126	0.8245	0.7045	0.9889	0.7598

**Table 6:** Performance Comparison between DTA-Unet and Three Image Processing Algorithms.

Method	MeanIoU	Precision	Recall	Accuracy	F-Measure
DTA-Unet	0.6126	0.8245	0.7045	0.9889	0.7598
Canny	0.0266	0.0403	0.0725	0.9336	0.0518
LOG	0.0093	0.0125	0.0358	0.9048	0.0185
Sobel	0.0410	0.0899	0.0702	0.9589	0.0789

**Table 7:** Performance Comparison between DTA-Unet and Other Deep Neural Network Models.

Model	MeanIoU	Precision	Recall	Accuracy	F-Measure
DTA-Unet	0.6126	0.8245	0.7045	0.9889	0.7598
FCN (VGG-based)	0.6016	0.8165	0.6956	0.9885	0.7513
Deeplab V3	0.5063	0.8279	0.5659	0.9862	0.6723

## Data Availability

The original contributions presented in this study are included in the article. Further inquiries can be directed to the corresponding author.
